# Multiparametric Magnetic Resonance Imaging Improves the Prognostic Outcomes in Patients With Intrahepatic Cholangiocarcinoma After Curative-Intent Resection

**DOI:** 10.3389/fonc.2022.756726

**Published:** 2022-03-09

**Authors:** Qian Li, Yi Wei, Feng Che, Tong Zhang, Shan Yao, Jian Zhao, YuHui Zhang, Hehan Tang, Bin Song

**Affiliations:** ^1^ Department of Radiology, Sichuan University West China Hospital, Chengdu, China; ^2^ Department of Evidence-Based Medicine and Clinical Epidemiology, West China Medical School of Medicine/West China Hospital, Sichuan University, Chengdu, China

**Keywords:** multiparametric magnetic resonance imaging, computed tomography, prognosis, intrahepatic cholangiocarcinoma, hepatectomy

## Abstract

**Purpose:**

The prognosis of patients with intrahepatic cholangiocarcinoma remains unclear. Thus, this study aimed at investigating whether additional multiparametric magnetic resonance imaging (mpMRI) would guide additional treatment and improve the prognostic outcomes of intrahepatic cholangiocarcinoma patients.

**Methods and Materials:**

This retrospective study included 256 patients undergoing dynamic enhanced computed tomography scan only (CT group) and 31 patients undergoing both mpMRI and computed tomography scans (CT+MR group). Propensity score matching (PSM) was used to minimize the potential selection bias and confounding effects. The overall survival (OS) and recurrence-free survival (RFS) rates were compared between the two groups.

**Results:**

More nodules (n = 6), additional biliary dilation (n = 4), and peritumoral parenchymal arterial phase hyperenhancement (n = 18) were found with the additional mpMRI scan, which led to treatment modification. Cox regression analysis revealed the survival advantage of additional mpMRI imaging based on the OS (HR 0.396, 95% CI 0.239–0.657, *p* < 0.001; PSM HR 0.400, 95% CI 0.218–0.736, *p* = 0.003) and RFS (HR 0.558, 95% CI 0.352–0.882, *p* = 0.013; PSM HR 0.508, 95% CI 0.288–0.897, *p* = 0.020).

**Conclusions:**

Additional mpMRI helps clinicians to select better treatment options, lower the risk of tumor recurrence, and improve the overall survival.

## 1 Introduction

Intrahepatic cholangiocarcinoma (ICC) accounts for approximately 10%–15% of cholangiocarcinoma cases and is the second most common primary liver cancer after hepatocellular carcinoma (HCC) (1). The incidence of ICC has also increased worldwide over the past decade (2). Unfortunately, due to non-specific symptoms, most patients are diagnosed at an advanced stage. Surgical resection remains the only potentially curative treatment (3). However, the recurrence rate after surgery is at 50%–70% (4, 5) and is directly related to a poor 5-year survival rate of 4%–35% (6). Adjuvant systemic treatments, such as capecitabine, have been reported to lower the recurrence rates and improve prognostic outcomes in patients with biliary tract cancer following radical resection; capecitabine has not been universally accepted as a novel standard of care because it failed to meet its primary endpoint of improving the overall survival (OS) in the intention-to-treat population. Further studies are necessary to better assess the role of adjuvant approaches in this aggressive malignancy (7–9). Meanwhile, preoperative risk factor assessment of ICC recurrence is vital for the precise management and prognostic improvement.

The early recurrence of ICC after surgery is partly attributed to the undetected occult lesion and the presence of a positive bile duct margin due to the extensive tumor extent of the disease (10). Therefore, accurate assessment of the overall tumor extent and detection of obscure lesions before surgery is of pivotal importance for clinical decision-making and improving clinical outcomes. Contrast-enhanced computed tomography (CECT) imaging is regarded as the standard imaging technique in diagnosis and guides the surgical planning for ICC because of its superior spatial resolution in evaluating the relationship between the tumor and its surrounding tissues, such as vessels and organs (11, 12). However, CECT also has the limitation that it may underestimate or even neglect the longitudinal tumor spread along the bile duct wall, especially for small lesions that only appear with bile duct thickening (13).

Magnetic resonance cholangiopancreatography (MRCP), a non-invasive magnetic resonance imaging (MRI) technique based on a heavily T2-weighted sequence, is superior in delineating the biliary extent of the tumor and providing an excellent overview of the overall biliary ducts proximal to the biliary obstruction (14, 15). Diffusion-weighted imaging (DWI), characterized by quantitative measurement of water diffusion in tissues, is important for evaluating tumor margins and detecting intrahepatic metastasis and lymph node metastasis (16). Moreover, dynamic contrast MRI is superior to CECT for the detection of small liver lesions, and it theoretically evaluates angioinvasion due to superior data acquisition and inherently greater contrast resolution (17–19). Based on the advantage of multiparametric imaging with MRI (mpMRI), it is helpful to detect bile duct invasion and occult intrahepatic metastasis and guide surgeons in making comprehensive surgical plans. Hence, it may further improve the prognosis of ICC after surgery. However, no study has investigated the prognostic value of additional mpMRI.

In this study, we aimed to determine whether the imaging findings of additional preoperative mpMRI would guide additional treatment and improve the prognosis of patients with ICC compared to those that only accept computed tomography (CT) examination.

## 2 Materials and Methods

### 2.1 Study Design and Patients

This retrospective study was conducted in accordance with the ethical guidelines of the Declaration of Helsinki and was approved by the institutional review board of West China Hospital, Sichuan University, and the requirement for written informed consent was waived. Participants with a pathohistological diagnosis of ICC were consecutively recruited from January 2009 to December 2017. The inclusion criteria were as follows: 1) patients aged ≥18 years; 2) patients with pathologically confirmed ICC; 3) less than 4-week interval between CECT, mpMRI, and curative-intent surgery (after considerate communication with patients about the cost and contradictions of mpMRI examinations and receiving the agreement of patients, mpMRI examinations were often used as supplementary means for CT examinations when CT screening was not sufficient in accurately determining liver tumor lesions); and 4) no preoperative anticancer treatment, such as transarterial chemoembolization or radiofrequency ablation. The exclusion criteria were as follows: 1) incomplete or poor quality of images and 2) patients who did not return for any clinical follow-up after surgery. Clinical data, including demographics, history of cirrhosis, Child–Pugh grade, outcomes of laboratory examination, operative method, extent of resection (surgical methods were assessed by preoperative multidisciplinary team discussion, and minor resections were defined as resection of three or less Couinaud segments; hemihepatectomy included mesohepatectomy, left hemihepatectomy, and right hemihepatectomy, and extended hepatectomy was defined as resection of five or more Couinaud segments), Roux-en-Y hepaticojejunostomy, and postoperative adjuvant therapy, were recorded from the electronic medical records system.

### 2.2 Imaging Acquisition

#### 2.2.1 CT Imaging

All patients underwent a multi-slice CT scan with four phases including unenhanced, arterial, portal venous, and delayed contrast-enhanced phase, using the following systems [LightSpeed VCT (GE Healthcare, Chicago, IL, USA), Sensation 64 CT (Siemens, Erlangen, Germany), or Sensation 16 CT (Siemens)] in West China Hospital. The scanning parameters were as follows: 100 or 120 kVp; tube current, 150–600 mA; slice thickness, 5 mm. After plain scanning was completed, a non-ionic contrast medium (iodine concentration, 370 mg/ml; volume, 1.5–2.0 ml/kg of body weight; contrast type, Iopromide injection, Bayer Pharma AG, Berlin, Germany) was injected at 3–5 ml/s through the antecubital vein, and 20 ml saline was injected after the injection of the contrast. Arterial phase, portal venous phase, and delayed phase scanning started at 30, 60, and 180 s after the contrast medium was injected.

#### 2.2.2 MR Imaging

The MRI scans of all the ICC patients were performed using a 3.0-T system (GE Healthcare; Siemens Healthcare). A 16-channel phased-array torso coil was used for all measurements. The mpMRI sequences included fast low-angle shot T1-weighted imaging in/out-of-phase, breath-hold fat-suppressed turbo spin-echo T2WI, MRCP, DWI, and dynamic multiphase enhanced imaging. The contrast agent (Omniscan, GE Healthcare) was injected with a dose of 0.2 ml/kg at a rate of 3 ml/s and then immediately followed by a flush of 30 ml saline. The images in the arterial phase, portal venous phase, and delayed phase were obtained at 30, 60, and 180 s after injection of the contrast agent. The detailed MRI scanning parameters are shown in **Table E1**.

### 2.3 Imaging Evaluation

All CT and MRI images were obtained and transferred to a workstation (Advantage Workstation 4.6; GE Medical Systems, Chicago, IL, USA). Two independent radiologists, who were blinded to the histopathological results, clinical data, and survival outcomes, reviewed all CT images. Two other abdominal radiologists, who were also blinded to the histopathological results, clinical data, liver CT imaging, and survival outcomes reviewed all MR images. Any discrepancies between the radiologists were discussed until a final consensus was reached.

The following imaging features were assessed by the above radiologists: 1) tumor morphology (well or ill); 2) tumor size, referring to the longest diameter of the lesion in the axial scan; 3) duct dilatation, referring to the encasement of the large (segmental or sectional) intrahepatic bile duct and/or peritumoral bile duct dilatation; 4) hepatic capsular retraction, referring to the invagination or focal flattening of the typical smooth contour of the liver capsule; 5) arterial phase enhancement pattern, referring to rim enhancement (rim-like enhancement that was pronounced in the tumor periphery), non-rim enhancement (arterial phase hyperenhancement of the non-rim pattern), or no/mild enhancement (diffuse hypoenhancement compared with liver parenchyma); 6) multifocality, whether more than one nodule; 7) satellite nodules, referring to tumors ≤2 cm in size and located ≤2 cm from the main tumor; 8) tumor in vein, referring to the unequivocal enhancing of the soft tissue in the vein, regardless of visualization of parenchymal mass; 9) central necrosis, in which the center of the tumor does not enhance at all or enhances very mildly at the arterial and portal venous phases; 10) portal venous phase washout, referring to hypointensity compared with the surrounding liver parenchyma on the portal venous phase; and 11) peritumoral parenchymal arterial phase hyperenhancement (APHE), referring to the grossly hyperarterial contrast material enhancement outside of the tumor border.

ICC was considered single when nodules close to the primary tumor were designated as satellite nodules; otherwise, ICCs were considered multiple. For patients with multiple tumors, all measurable observations were assessed and the largest observation was selected as the representative for statistical analysis.

### 2.4 Pathological Evaluation

Another researcher who did not take part in the imaging evaluation independently searched the pathological information of ICC patients in the electronic medical record system. Pathologic parameters, including tumor size of the resection sample, pathologic TNM staging (stages Ia, Ib, II, IIIa, IIIb, and IV) according to the eighth edition of AJCC staging, tumor differentiation, lymph node metastasis, and microvascular invasion, were collected.

### 2.5 Follow-Up Evaluation

The routine follow-up protocol included detection of serum tumor markers and imaging studies, including ultrasonography, CT, and/or MRI at intervals of 3–6 months after surgery. Patients were followed up from the index date of surgery to death or to the last follow-up date (December 30, 2020). OS was measured as the interval from the date of surgery to death from a disease-related cause. Recurrence-free survival (RFS) was measured from the date of surgery to the date of tumor recurrence. For patients who were alive at the last follow-up, OS was measured from the date of surgery to the date of last contact. Similarly, for patients who did not experience recurrence during the last assessment period, RFS was measured from the date of surgery to the date of last contact.

### 2.6 Statistical Analysis

All patients who met the inclusion criteria were included in the analysis. The categorical variables in the baseline data were shown as frequencies and proportions, and the continuous variables are described as mean and standard deviation or median and interquartile range (IQR), as appropriate. The characteristics of the two groups (CT group and CT+MRI group) were compared using an unpaired t-test or Mann–Whitney *U* test for continuous variables, and the chi-square test or Fisher’s exact test for categorical variables, respectively. The changes in imaging findings in the CT+MRI group were compared using the paired t-test for continuous variables and the McNemar test for categorical variables. The survival curves of OS and RFS were drawn using the Kaplan–Meier method, and the differences in OS and RFS between the two groups were compared using the log-rank test. Variables which were significant on univariate analysis (*p* < 0.05) were subsequently included in the multivariable Cox proportional hazard model. To minimize the effect of selection bias and potential confounders between the two groups, propensity score matching (PSM) with a ratio of 1:1 nearest matching was used for the sensitivity analysis (20), and the caliper was set at 0.10. Considering that variables related to the exposure or related to the outcome should be measured and included in the propensity score model (21), from a clinical perspective, the propensity score was estimated by applying a multivariable logistic regression model with eight adjustment variables: age, gender, cirrhosis, cancer antigen 199, carcinoma embryonic antigen, tumor size on CT images, multiple tumors on CT images, and satellite nodules on CT images (22, 23). The interobserver agreement was applied to assess the reliability of imaging analysis using the Kappa test; 0–0.2, slight; 0.21–0.40, fair; 0.41–0.60, moderate; 0.61–0.80, substantial; and 0.81–1, excellent.

All statistical analyses were performed using SPSS (version 25, IBM), along with the R software (version 4.0.2, http://www.R-project.org/) with packages “MatchIt,” “tableone,” “Matching,” “reshape2,” “reportReg,” “survminer,” and “survival.” Statistical significance was set at *p* < 0.05 (two-sided).

## 3 Results

### 3.1 Basic Characteristics of Included Subjects

**Figure 1:**
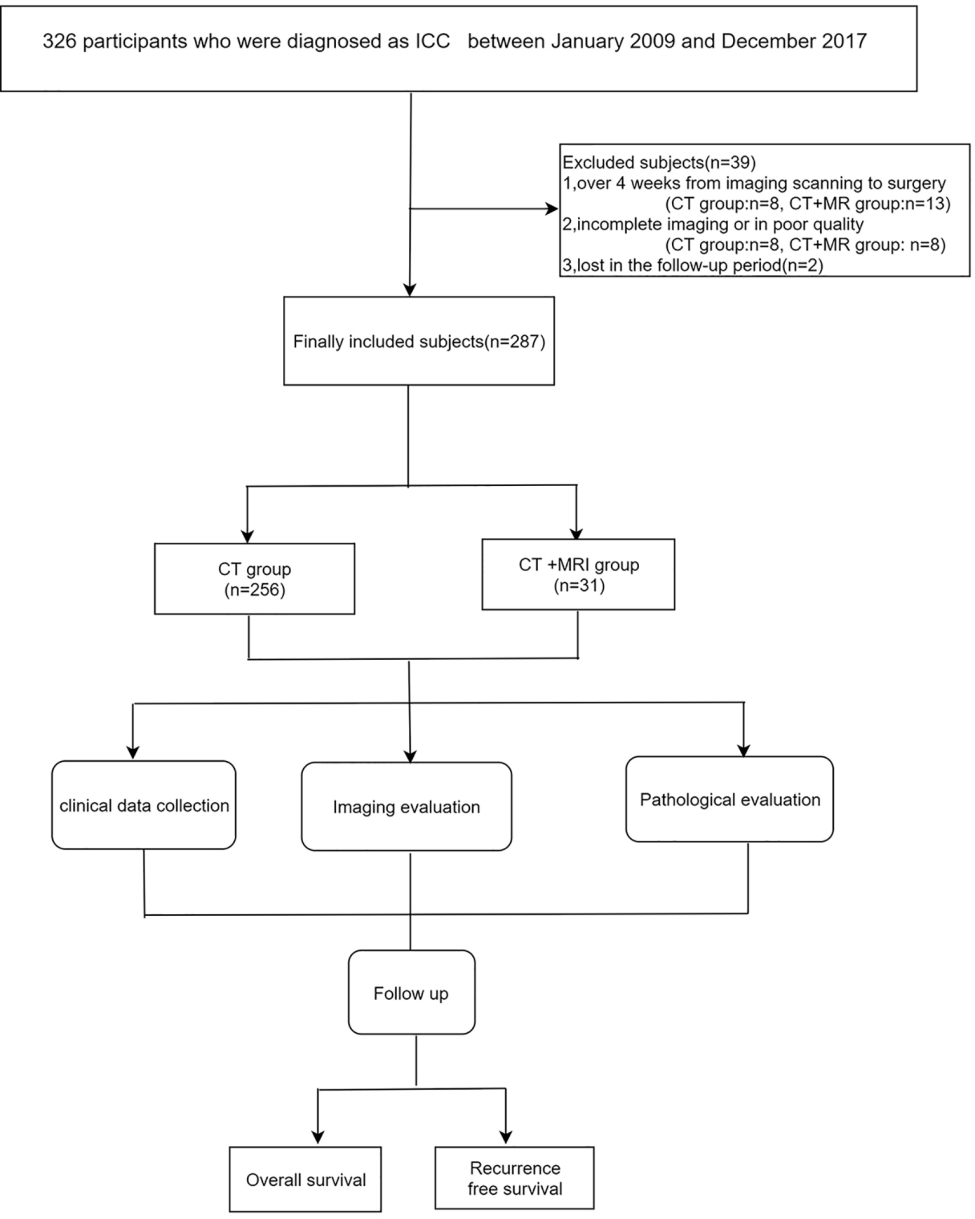
Flow diagram of this study.

As shown in **Figure 1**, 287 patients with ICC met the inclusion criteria and were finally enrolled in our study, of whom 256 patients underwent CECT scan only (CT group) and 31 underwent both CECT and mpMRI scans (CT+MR group). As shown in **Tables 1**, **2**, most baseline characteristics were comparable between the CT and CT+MRI groups, for instance, the percentage of Child–Pugh grade A (96.88% vs. 96.77%, *p* = 0.976), microvascular invasion (10.55% vs. 3.23%, *p* = 0.681), and multifocality evaluated by CT imaging (5.47% vs. 9.68%, *p* = 0.714). However, ICC patients in the CT+MRI group were significantly younger than those in the CT group (53.35 ± 10.59 years vs. 57.64 ± 10.18 years, *p* = 0.028), and most satellite nodules manifested in the CT group (33.98% vs. 16.13%, *p* = 0.034). In addition, the Kappa test showed substantial performance (mean, 0.756; range, 0.560–0.890) for imaging feature evaluation.

**Table 1:** Demographic and clinical characteristics of the ICC patients.

Factors	CT group (*n* = 256)	CT+MRI group (*n* = 31)	*p* value
Age (years)	57.64 ± 10.18	53.35 ± 10.59	0.028*^a^*
Gender (female %)	115 (44.92)	18 (58.06)	0.166
Cirrhosis (%)	72 (28.13)	9 (29.03)	0.916
CA19-9 (>37 u/ml, %)	169 (66.02)	16 (51.61)	0.114
AFP (>20 ng/ml, %)	20 (7.81)	3 (9.68)	0.724
CEA (>5 ng/ml, %)	70 (27.34)	6 (19.35)	0.341
HBsAg (%)	81 (31.64)	8 (25.81)	0.318
HBeAg (%)			
Tumor differentiation (%)			0.604
Well	7 (2.73)	1 (3.23)	
Moderate-poor	249 (97.27)	30 (96.77)	
LNM (%)	66 (25.78)	6 (19.35)	0.329
MVI (%)	27 (10.55)	1 (3.23)	0.681
Tumor size on resection sample (cm)	6.13 ± 2.39	6.08 ± 2.85	0.921
Child–Pugh grade (%)			0.976
A	248 (96.88)	30 (96.77)	
B	8 (3.13)	1 (3.23)	
AJCC stage (%)			0.832
IA	29 (11.33)	4 (12.9)	
IB	17 (6.64)	0 (0)	
II	26 (10.16)	5 (16.13)	
IIIA	115 (44.92)	16 (51.61)	
IIIB	69 (26.95)	6 (19.35)	
Roux-Y hepaticojejunostomy (%)	54 (21.09)	11 (35.48)	0.071
Type of surgical resection (%)			0.836
Minor resections	56 (21.88)	6 (19.35)	
Hemihepatectoy	138 (53.91)	16 (51.61)	
Extended hepatectomy	62 (24.22)	9 (29.03)	
Positive resection margin (%)	17 (6.64)	1 (3.23)	0.704
Postoperative adjuvant therapy (%)	40 (15.63)	9 (29.03)	0.061

Data are represented in mean ± SD or frequency (%). Data were evaluated by independent t test or Mann–Whitney U test for continuous variables and the chi-square test or Fisher’s exact test for categorical variables.

a p < 0.05.

CA199, cancerantigen199; AFP, alpha-fetoprotein; CEA, carcinoma embryonic antigen; INR, international normalized ratio; FIB, plasma fibrinogen; HbsAg, hepatitis B surface antigen; MVI, microvascular invasion; LNM, lymph node metastasis; AJCC, American Joint Committee on Cancer.

**Table 2:** Imaging findings based on CT of the ICC patients.

Imaging findings based on CT	CT group (*n* = 256)	CT+MRI group (*n* = 31)	*p* value
Size (cm)	6.29 ± 2.42	6.14 ± 2.66	0.760
Ill border (%)	242 (94.53)	28 (90.32)	0.383
Peritumoral liver capsule retraction (%)	16 (6.25)	2 (6.45)	0.965
Multifocality (%)	14 (5.47)	3 (9.68)	0.714
Satellite nodule (%)	87 (33.98)	5 (16.13)	0.034*^a^*
Central necrosis (%)	74 (28.91)	7 (22.58)	0.598
Biliary obstruction (%)	104 (40.63)	16 (51.61)	0.241
Tumor in vein (%)	74 (28.91)	10 (32.26)	0.698
Peripheral APHE (%)	47 (18.36)	6 (19.35)	0.879
AP enhancement (%)			0.545
Rim	109 (42.58)	15 (48.39)	
Non-rim	143 (55.86)	16 (51.61)	
Non-enhancement	4 (1.56)	0 (0)	
PVP washout (%)			0.204
Washout	14 (5.47)	0 (0)	
No washout	242 (94.53)	31 (100)	

Data are represented in mean ± SD or frequency (%). Data were evaluated by independent t test or Mann–Whitney U test for continuous variables and the chi-square test or Fisher’s exact test for categorical variables.

a Referred to p < 0.05.

AP, arterial phase; PVP, portal venous phase;APHE, peripheral arterial phase hyperenhancement.

### 3.2 Changes in Imaging Findings Evaluated by mpMRI in the CT+MRI Group

In the CT+MRI group (**Table 3**), additional 6 patients (*p* = 0.031) were found to have additional occult tumors (all tumor size <2 cm) using mpMRI. Intriguingly, larger tumor sizes were observed in 24 patients (77.42%) (6.12 ± 2.72 cm vs. 5.91 ± 2.51 cm, *p* = 0.041) based on mpMRI compared with those evaluated by CT. Peritumoral parenchymal APHE was also found in additional 18 patients (*p* < 0.001).The presence of biliary dilation manifested in additional 4 ICC patients using MRCP of mpMRI.

**Table 3:** Changes in imaging findings based on MRI of the ICC patients.

Patient, *n* (%)	Total	Number of ICC patients showing biliary dilation by CECT		
		Yes	No	*p* value
Number of ICC patients showing biliary dilation by mpMRI	20	16 (80.00%)	4 (20.00%)	0.125
Patient, *n* (%)		Number of ICC patients showing more than one nodule by CECT		
	Total	Yes	No	*p* value
Number of ICC patients showing more than one nodule by mpMRI	14	8 (57.14%)	6 (42.86%)	0.031*^a^*
Patient, *n* (%)		Number of ICC patients showing peritumor APHE by CECT		
	Total	Yes	No	*p* value
Number of ICC patients showing peritumor APHE by mpMRI	24	6 (25.00%)	18 (75.00%)	<0.001*^a^*
Imaging findings	CECT	mpMRI	Difference	*p* value
Tumor size (cm)	6.12 ± 2.72	5.91 ± 2.51	0.21 ± 0.72	0.041*^a^*

Data are represented in mean ± SD or frequency. Data were evaluated by paired t test for continuous variables and McNemar test for categorical variables.

a p < 0.05.

APHE, peripheral arterial phase hyperenhancement; Yes referred to that the imaging findings based on mpMRI were also found in CECT imaging, while No referred to that the imaging findings based on mpMRI cannot be found in CECT imaging.

### 3.3 Treatment Options to the Additional Imaging Findings After mpMR Imaging

In the CT+MRI group (**Figure E1**), for patients with more than one tumor nodule only identified by mpMRI, 3 patients finally underwent extended liver resection with a negative resection margin, and two underwent hemihepatectomy with a negative resection margin; the primary and additional nodules were simultaneously covered in the resection extent, and the remaining one received radiofrequency ablation for the additional lesion within 1 month after the primary hepatectomy for the primary nodule. The tumor sizes on mpMRI (MRI: R^2^ = 0.980, *p* < 0.001; CT: R^2^ = 0.856, *p* < 0.001) were more relevant to those on pathological confirmation (**Figure E2**). Moreover, for patients with peritumoral parenchymal APHE only manifesting in mpMRI, 4 patients finally underwent extended liver resection with a negative resection margin, 10 underwent hemihepatectomy with negative resection margin, and 4 patients underwent minor resection with a negative resection margin, whose resection extent all included peritumoral parenchyma showing APHE. The 4 patients with additional biliary dilation manifested only in mpMRI all underwent Roux-en-Y hepaticojejunostomy.

### 3.4 Survival Outcomes

The median follow-up period was 56.6 months (IQR 43.2–64.5 months) in the CT group, which was similar (*p* = 0.258) to that in the CT+MRI group (52.9 months, IQR 43.8–64.0 months); 254 (88.50%) patients manifested tumor recurrence and 242 (84.32%) patients died.

**Figure 2:**
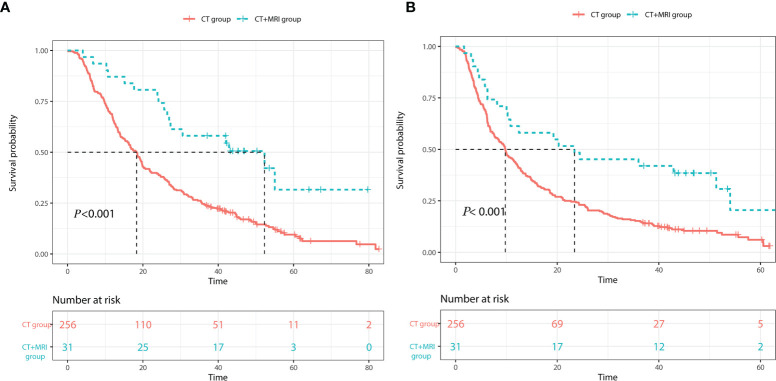
Kaplan–Meier curves of **(A)** overall survival and **(B)** recurrence-free rates of the CT group and CT+MRI group.

According to the survival curves (**Figure 2A**), the 1-, 3-, and 5-year survival rates of the CT group and CT+MRI groups were 66.80%, 24.61%, 4.30% and 87.10%, 58.06%, 9.67%, respectively. The OS rate in the CT+MRI group was significantly higher than that in the CT group (*p* < 0.001). In addition, the 1-, 2-, and 3-year RFS rates of the CT and CT+MRI groups were 42.97%, 24.22%, 15.23% and 61.29%, 48.38%, 45.16%, respectively. The CT+MRI group (**Figure 2B**) achieved the greater RFS rates (*p* < 0.001). Moreover, in the multivariable Cox proportional hazard model, the CT+MR group was an independent prognostic factor for a lower risk of mortality and recurrence (OS: HR 0.396, 95% CI 0.239–0.657, *p* < 0.001; RFS: HR 0.558, 95% CI 0.352–0.882, *p* = 0.013) (**Table 4**).

**Table 4:** Cox regression analysis of OS and RFS for patients with ICC.

	OS				RFS				
Factors	Univariate analysis		Multivariable analysis		Univariate analysis		Multivariable analysis		
	HR (95% CI)	*p*value	HR (95% CI)	*p* value	HR (95% CI)	*p*value	HR (95% CI)	*p*value	
Group									
CT	Reference		Reference		Reference		Reference		
CT+MRI	0.390 (0.238,0.639)	<0.001*^a^*	0.396 (0.239,0.657)	<0.001*^a^*	0.473 (0.301,0.741)	0.001*^a^*	0.558 (0.352,0.882)		0.013*^a^*
CA199 (>37 u/ml)	1.439 (1.099,1.884)	0.008*^a^*	1.211 (0.912,1.608)	0.186	1.382 (1.065,1.794)	0.015*^a^*	–		
AFP (>20 ng/ml)	1.214 (0.768,1.919)	0.407			1.369 (0.874,2.143)	0.170			
CEA (>5 ng/ml)	1.629 (1.232,2.155)	0.001*^a^*	1.384 (1.028,1.864)	0.032*^a^*	1.501 (1.138,1.979)	0.004*^a^*	1.379 (1.039,1.830)		0.026*^a^*
HBsAg	1.149 (0.875,1.508)	0.318			1.109 (0.850,1.447)	0.445			
HesAg	1.235 (0.580,2.630)	0.585			1.712 (0.845,3.469)	0.136			
Age	0.999 (0.987,1.011)	0.814			0.997 (0.985,1.009)	0.625			
Gender (female)	0.696 (0.54,0.897)	0.005*^a^*	0.724 (0.553,0.948)	0.019*^a^*	0.805 (0.629,1.031)	0.086			
Cirrhosis	1.031 (0.774,1.373)	0.836			0.839 (0.631,1.115)	0.226			
Tumor size on resection sample	1.038 (0.985,1.093)	0.160			1.044 (0.993,1.098)	0.089			
Tumor differentiation									
Well	Reference				Reference		–		
Moderate-poor	1.970 (0.812,4.782)	0.134			2.520 (1.038,6.120)	0.041*^a^*	–		
LNM	2.455 (1.84,3.275)	<0.001*^a^*	0.875 (0.271,2.827)	0.823	2.186 (1.651,2.893)	<0.001*^a^*	–		
MVI	2.025 (1.349,3.039)	0.001*^a^*	1.416 (0.928,2.159)	0.107	2.198 (1.465,3.298)	<0.001*^a^*	1.632 (1.075,2.477)		0.021*^a^*
Child–Pugh									
A	Reference								
B	1.239 (0.611,2.511)	0.552			1.339 (0.687,2.611)	0.392			
AJCC stage		<0.001*^a^*		0.375		<0.001*^a^*			<0.001*^a^*
IA	Reference		Reference		Reference		Reference		
IB	2.200 (1.153,4.196)	0.017	1.505 (0.778,2.911)	0.224	2.329 (1.227,4.422)	0.010	1.869 (0.978,3.570)		0.058
II	1.155 (0.646,2.065)	0.626	1.153 (0.642,2.071)	0.633	1.305 (0.744,2.287)	0.353	1.283 (0.729,2.259)		0.388
IIIA	1.206 (0.762,1.908)	0.425	1.122 (0.706,1.784)	0.625	1.498 (0.956,2.346)	0.078	1.439 (0.916,2.259)		0.114
IIIB	3.007 (1.857,4.869)	<0.001	2.976 (0.882,10.048)	0.079	3.069 (1.91,4.932)	<0.001	2.674 (1.652,4.328)		<0.001*^a^*
AP enhancement on CT imaging		0.505				0.406			
Rim	Reference				Reference				
Non-rim	0.904 (0.7,1.168)	0.441			0.864 (0.673,1.109)	0.250			
Non-enhancement	1.476 (0.543,4.012)	0.445			1.311 (0.483,3.558)	0.595			
Tumor size on CT imaging	1.942 (0.990,1.096)	0.119			1.047 (0.996,1.101)	0.070			
Multifocality on CT imaging	0.644 (0.392,1.058)	0.082			0.822 (0.513,1.315)	0.413			
Satellite nodule on CT imaging	1.856 (1.424,2.418)	<0.001*^a^*	1.713 (1.299,2.259)	<0.001*^a^*	1.699 (1.311,2.203)	<0.001*^a^*	1.577 (1.210,2.054)		0.001*^a^*
Biliary obstruction on CT imaging	1.232 (0.955,1.59)	0.109			1.165 (0.908,1.493)	0.229			
APHE on CT imaging	1.327 (0.964,1.826)	0.103			1.181 (0.864,1.614)	0.291			
Ill border on CT imaging	1.887 (0.925,3.472)	0.061			1.932 (0.079,3.461)	0.627			
Internal artery on CT imaging	1.031 (0.799,1.331)	0.812			1.115 (0.87,1.429)	0.389			
PVP no washout on CT imaging	0.222 (0.054,1.008)	0.106			0.263 (0.065,1.066)	0.101			
Delayed central enhancement on CT imaging	1.103 (0.601,2.022)	0.752			1.114 (0.608,2.041)	0.726			
Capsule on CT imaging	0.557 (0.307,1.012)	0.055			1.137 (0.684,1.888)	0.621			
Central necrosis on CT imaging	1.069 (0.811,1.41)	0.635			1.182 (0.904,1.545)	0.221			
Tumor in vein on CT imaging	1.169 (0.888,1.54)	0.265			1.221 (0.934,1.597)	0.144			

a p < 0.05.

CA199, cancer antigen 199; AFP, alpha-fetoprotein; CEA, carcinoma embryonic antigen; INR, international normalized ratio; FIB, plasma fibrinogen; HBsAg, hepatitis B surface antigen; MVI, microvascular invasion; LNM, lymph node metastasis; AJCC, American Joint Committee on Cancer; AP, arterial phase; APHE, peripheral arterial phase hyperenhancement; PVP, portal venous phase.

### 3.5 Propensity Score Matching

After the propensity score matching, 31 matched pairs of ICC patients from the CT and CT+MRI groups were selected. The baseline characteristics of the demographic and clinical data, and imaging findings in the two groups, were balanced (all *p* > 0.05, **Tables E2**, **E3 **and** Figure E3**).

**Figure 3:**
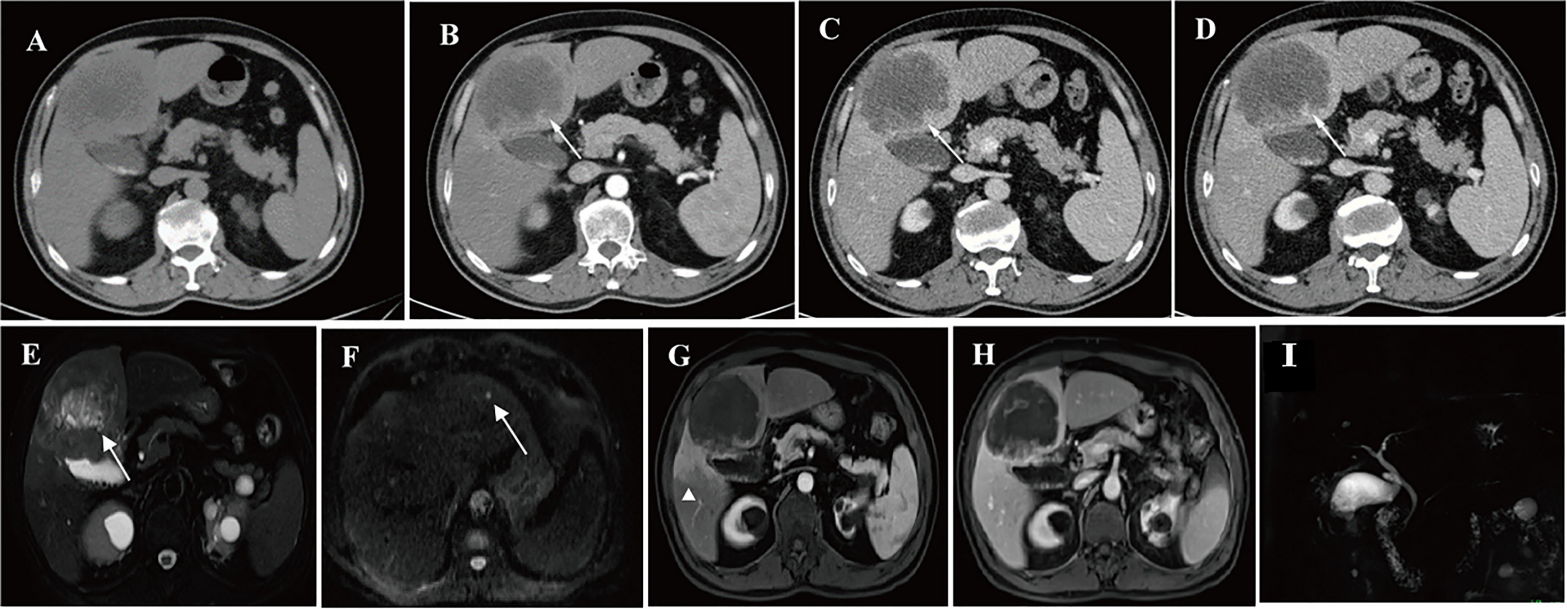
CECT and mpMRI of a representative patient: this 62-year-old man was initially assessed as having a single nodule of intrahepatic cholangiocarcinoma by CECT, while an additional probable nodule was found by MR images with DWI. Pathological examination confirmed that the additional nodule was also ICC, the patients finally received extended liver resection to remove the primary and the secondary tumor module. **(A–D)** indicate a nodule showing typical features of ICC (rimenhancement in the arterial phase and no washout in the portal venous phase and delayed phase) in dynamic CT images (arrow): **(A)** pre-contrast phase; **(B)** arterial phase; **(C)** portal phase; and **(D)** delayed phase. **(E–I)** show dynamic MRI images with DWI and MRCP: **(E)** shows the same ICC nodule that was detected with DWI (arrow); **(F)** shows a probable ICC nodule that were found by DWI (arrow); **(G)** shows peritumor parenchymal arterial phase alteration (arrowhead) and no washout in portal venous phase **(H)**; and no biliary dilation displays in **(I)**.

When considering OS and RFS in the matched data, the CT+MRI group showed better prognostic outcomes than the CT group (**Figure E4**) (OS: HR 0.400, 95% CI 0.218–0.736, *p* = 0.003; RFS: HR 0.508, 95% CI 0.288–0.897, *p* = 0.020). **Figure 3** showed a representative patients who showed additional nodule with mpMRI scan compared to only CT scan and received additional treatment to improve prognosis.

## 4 Discussion

To the best of our knowledge, this is the first study to compare the prognostic outcomes of ICC patients undergoing CECT scan only with those undergoing both CECT and mpMRI scans. Our results showed that mpMRI significantly improved RFS and OS of ICC patients compared with CECT scan in univariate, multivariate, and propensity score matched analyses. More occult lesions, larger tumor size, peritumoral parenchymal APHE lesions, and additional biliary dilation were found using mpMRI, which were in accordance with additional treatment, such as extended resection extent including additional nodules and peritumoral parenchymal showing APHE, radiofrequency ablation, and Roux-en-Y hepaticojejunostomy.

As part of the AJCC/UICC staging system, tumor size and number are two indispensable prognostic factors in patients with ICC (24). In our study, more nodules were detected using mpMRI, which was in accordance with previous studies that MRI enabled to detect more additional focal liver lesions than CT (18, 25). This may be due to the superior high soft tissue resolution of MR imaging. Moreover, the use of diffusion-weighted imaging can also further improve the lesion detectability (25, 26). All additional nodules found only by mpMRI were removed through surgical resection (extended liver resection or hemihepatectomy) or radiofrequency ablation. Without the additional mpMRI scan, these additional nodules not suspected on CT images might not receive timely treatment and appear as recurrences. Similar to our study, Gaya et al. (27) also found that ICC patients with multifocality (≥2) underwent an extended hepatectomy more frequently to remove all nodules and lower the presence of tumor recurrence. Surgical resection plus radiofrequency ablation has also been proven to be a favorable treatment for multifocal intrahepatic cholangiocarcinoma with sufficient future liver remnant and achieved a better 1-year OS rate than surgery only or radiation only (28). Therefore, we consider that mpMRI is a more sensitive imaging modality to evaluate tumor nodules and help surgeons to make appropriate treatment plans for all lesions to achieve better prognosis. In our study, the larger tumor size using mpMRI was closer to that from pathology measurements in comparison with CECT, and this may be attributed to the insufficient contrast between tumor and adjacent soft tissue on CECT scan (29).

Our results found that mpMRI could find more peripheral APHE lesions compared with CECT. Peritumoral stromal reaction is indispensable in the ICC carcinogenesis process; as the tumor gradually invades into the peritumoral stroma/stromal vasculature and replaces adjacent hepatic cells, the occult minute invasion and vessel invasion already appear and display as peritumoral parenchymal arterial phase hyperenhancement (30). In comparison with CECT, contrast-enhanced MRI has been proven to be more sensitive, for that the contrast could enter the extravascular or extracellular compartment, and it can provide more information about microvessel perfusion, permeability, and extracellular leakage space (31). On the other hand, ICC tumor cells originate from the epithelial lining of the intrahepatic bile duct. Hence, tumors enable the occlusion of the intrahepatic bile duct and cause peripheral cholangitis, which may also manifest as peritumoral parenchymal APHE (32). However, there is no effective preoperational assessment method to verify what it is indeed, and postoperative surgical specimens, including the lesion showing APHE, are a prerequisite to ensuring the integrity of pathological data. Hence, APHE works as a noteworthy hint for the extent of liver resection and guides clinicians in developing the next treatment plan based on the final pathological results, which may improve the prognosis and lower the risk of recurrence.

In our study, compared with CT, mpMRI including MRCP was more sensitive in detecting obstructive bill ducts. Similar findings have been reported in recent studies (33, 34).The presence of biliary dilation has been proven to be associated with large bill duct ICC, which was characterized by low cellularity and abundant fibrous stroma, and a poor prognostic outcome (35). Moreover, intrahepatic bile duct dilatation indeed indicates the presence of intrahepatic cholangiocarcinoma (36, 37). For ICC patients with biliary obstruction, Roux-en-Y hepaticojejunostomy was the standard treatment (10). In our study, the additional biliary dilation found by the sensitive imaging modality-mpMRI guided the patients into receiving the above treatment options in time, which might also be noteworthy for prognostic improvement.

This real-world study proved that mpMRI led to the additional treatment and significantly improved clinical outcomes of ICC patients compared with those who underwent CECT scan only. This study has some clinical implications. First, it may provide clinicians with clinical evidence that additional mpMRI before treatment may assist more precise and individual management of ICC patients and improve overall prognosis. In addition, this study may help clinical institutions to optimize their general clinic pathway for ICC precise treatment and management. For instance, mpMRI examination could be used as an additional alterative imaging modality for those patients suspected of having ICC but not only confined in CT imaging. The obvious difference in sample size between the CT and CT+MRI groups was associated with the instability of results, which was the biggest knowledge gap of this present study. Accordingly, we performed a PSM analysis with one-to-one match to minimize its influence.

This study has several limitations. First, the retrospective design based on a single research institution may cause selection bias and limit its feasibility. However, propensity score matching (PSM) was performed, which may have helped to minimize the systemic and statistical bias and simulate a random controlled trial. Second, no patient with gadoxetic acid-enhanced MRI was included, despite the use of DWI which was also identified as excellent sequences for the detection of small lesions (38). Gadoxetic acid-enhanced MRI imaging has proven to be a useful tool for identifying small lesions (<2 cm) and improving the prognostic outcome. Thus, in our further study, a prospective, multicenter study that collects data of patients who undergo gadoxetic acid-enhanced MRI scan should be conducted. Finally, a CT scan with a thinner slice thickness, like 1.2 mm, may lead to a more detailed assessment of small hepatic lesions. However, the most effective slice thickness for liver imaging remains uncertain (39). In clinical practice, a 5-mm CT thickness on contrast-enhanced CT imaging is regarded as the routine slice thickness for patients who are suspected of having focal liver lesions such as hepatocellular carcinoma or ICC. However, our study is a real-world setting, which aims at producing real-world evidence that provides adequate scientific evidence for regulatory decision-making, and further study about whether CT image thickness would influence the prognostic outcomes should be conducted.

## 5 Conclusion

In conclusion, our study found that mpMRI contributed to the additional detection of more occult lesions, larger tumor size, peritumor parenchymal alteration, and biliary dilation. The above additional imaging findings may help surgeons choose appropriate treatment options, further improving the OS and RFS of patients with ICC.

## Data Availability Statement

The original contributions presented in the study are included in the article/**Supplementary Material**. Further inquiries can be directed to the corresponding authors.

## Ethics Statement

The studies involving human participants were reviewed and approved by the institutional review board of West China Hospital, Sichuan University. Written informed consent for participation was not required for this study in accordance with the national legislation and the institutional requirements.

## Author Contributions

QL and YW had full access to all of the data in the study and take responsibility for the integrity of the data and the accuracy of the data analysis. QL and YW contributed equally to the study. Concept and design: QL, YW, HT, and BS. Acquisition, analysis, or interpretation of data: all authors. Drafting of the manuscript: QL and YW. Critical revision of the manuscript for important intellectual content: all authors. Statistical analysis: QL, YW, and YZ. Obtained funding: BS. Administrative, technical, or material support: all authors. Supervision: BS. All authors contributed to the article and approved the submitted version.

## Funding

BS received grants from the National Natural Science Foundation of China (Nos. 81771797, 81971571) and Science and Technology Support Program of Sichuan Province (No.2021YFS0021). YW received grants from China Postdoctoral Science Foundation (No. 2021M692289). All the funds are from the government, BS and YW have no conflicts to disclose.

## Conflict of Interest

The authors declare that the research was conducted in the absence of any commercial or financial relationships that could be construed as a potential conflict of interest.

The reviewer HW declared a shared affiliation, with the authors to the handling editor at the time of the review.

## Publisher’s Note

All claims expressed in this article are solely those of the authors and do not necessarily represent those of their affiliated organizations, or those of the publisher, the editors and the reviewers. Any product that may be evaluated in this article, or claim that may be made by its manufacturer, is not guaranteed or endorsed by the publisher.
