# Supplementary material for: Multiparametric Magnetic Resonance Imaging Improves the Prognostic Outcomes in Patients With Intrahepatic Cholangiocarcinoma After Curative-Intent Resection
Source: Front Oncol. 2022 Mar 9;12:756726. doi: 10.3389/fonc.2022.756726 (PMC8959855; doi:10.3389/fonc.2022.756726)

Table E1 MRI sequences and parameters

| Sequence | Repetition time(ms) | Echo time(ms) | Field of View (cm^2)^ | Scan matrix | Slice thickness(mm) | Motion compensation | Fat saturation |
| --- | --- | --- | --- | --- | --- | --- | --- |
| VIBE | 4.03 | 1.43 | 42*25 | 320*192 | 4 | Breath hold | Yes |
| MRCP | 4500 | 707 |  | 348*346 | 40 | Respiratory trigger | Yes |
| T1WI | 181 | 2.2 | 45*23 | 256*106 | 6 | Breath hold | None |
| T2WI | 4480 | 102 | 42*26 | 320*157 | 6 | Respiratory trigger | Yes |
| DWI | 6000 | 73 | 39*28 | 192*110 | 6 | Respiratory trigger | Yes |

b=0、50、500、800、1000 s/mm2; VIBE, volume interpolated breath-hold examination; Vibe, volume interpolated breath-hold examination; T1WI, T1-weighted imaging; MRCP, Magnetic Resonance Cholangio-pancreatography; T2WI, T2-weighted imaging; DWI, Diffusion-weighted imaging.

Table E2 Demographic and clinical characteristics of the ICC patients afte Propensity Score Matching

| Factors |  | Propensity Score-Matching | | |
| --- | --- | --- | --- | --- |
|  |  | CT group  (*n*=31) | CT+MRI group(*n*=31) | *P* value |
| Age (years) |  | 54.55 (11.42) | 53.35 (10.59) | 0.671 |
| Gender (female %) |  | 15(48.39%) | 13(41.94%) | 0.610 |
| Cirrhosis (%) |  | 7(22.58%) | 9(29.03%) | 0.562 |
| CA199(>37u/ml %) |  | 19(61.29%) | 16(51.61%) | 0.442 |
| AFP (>20 ng/ml %) |  | 1(3.23%) | 3(9.68%) | 0.612 |
| CEA (>5ng/ml %) |  | 6(19.35%) | 6(19.35%) | 1.000 |
| HBsAg (%) |  | 12(38.71%) | 8(25.81%) | 0.277 |
| HBeAg (%) |  | 1(3.23%) | 0(0%) | 1.000 |
| Tumor differentiation |  |  |  | 1.000 |
| Well |  | 0(0.00%) | 1(3.23%) |  |
| Moderate-poor |  | 31(100%) | 30(96.77%) |  |
| LNM (%) |  | 8(25.81%) | 6(19.35%) | 0.544 |
| MVI (%) |  | 4(12.9%) | 1(3.23%) | 0.354 |
| Tumor size on resection sample |  | 5.57 (2.51) | 6.08 (2.85) | 0.455 |
| Child Hugh grade (%) |  |  |  | 1.000 |
| A |  | 30(96.77%) | 30(96.77%) |  |
| B |  | 1(3.23%) | 1(3.23%) |  |
| AJCC/UICC stage (%) |  |  |  | 0.564 |
| IA |  | 3(9.68%) | 4(12.90%) |  |
| IB |  | 2(6.45%) | 0(0.00%) |  |
| II |  | 3(9.68%) | 5(16.13%) |  |
| IIIA |  | 15(48.39%) | 16(51.61%) |  |
| IIIB |  | 8(25.80%) | 6(19.35%) |  |
| Roux‐Y hepaticojejunostomy (%) |  | 12(38.71%) | 11(35.48%) | 0.793 |
| Type of surgical resection (%) |  |  |  | 0.941 |
| Minor resections |  | 5(16.13%) | 6(19.35%) |  |
| Hemihepatectomy |  | 17(54.84%) | 16(51.61%) |  |
| Extended hepatectomy |  | 9(29.03)5 | 9(29.03%) |  |
| Positive resection margin(%) |  | 1(3.23%) | 1(3.23%) | 1.000 |
| Postoperative adjuvant therapy (%) |  | 8(25.81%) | 8(25.81%) | 1.000 |

NOTE: Data are represented in mean ± SD or frequency (%). And Data were evaluated by independent *t* test or Mann-Whitney *U* test for continuous variables and the Chi-square test or Fisher’s exact test for categorical variables; CA199, cancerantigen199; AFP, alpha-fetoprotein; CEA, Carcinoma Embryonic Antigen; INR, International Normalized Ratio; FIB, [plasma](javascript:;) [fibrinogen](javascript:;); HBsAg, Hepatitis B surface antigen; MVI, Microvascular invasion; LNM, lymph node metastasis

Table E3 Imaging findings based on CT of the ICC patients after Inverse Probability Weighting and Propensity Score Matching

| Imaging findings |  | Propensity Score-Matching | | |
| --- | --- | --- | --- | --- |
|  |  | CT group  (*n*=31) | CT+MRI  group (*n*=31) | *P* value |
| Size (cm) |  | 5.87 (2.66) | 6.14 (2.66) | 0.691 |
| Ill border (%) |  | 30(96.77%) | 28(90.32%) | 0.612 |
| Internal artery (%) |  | 11(35.48%) | 14(45.16%) | 0.437 |
| Capsule (%) |  | 3(9.68%) | 2(6.45%) | 1.000 |
| Multifocality (%) |  | 5(16.13%) | 3(9.68%) | 0.857 |
| Satellite nodule (%) |  | 2(6.45%) | 5(16.13%) | 0.425 |
| Central necrosis (%) |  | 8(25.81%) | 7(22.58%) | 0.767 |
| Biliary obstruction (%) |  | 11(35.48%) | 16(51.61%) | 0.200 |
| APHE (%) |  | 4(12.9%) | 6(19.35%) | 0.409 |
| Tumor in vein (%) |  | 5(16.13%) | 10(32.26%) | 0.138 |
| AP enhancement(%) |  |  |  | 0.597 |
| Rim |  | 15(48.39%) | 15(48.39%) |  |
| Non-rim |  | 15(48.39%) | 16(51.61%) |  |
| Nonenhancement |  | 1(3.22%) | 0(0.00%) |  |
| PVP washout (%) |  |  |  |  |
| Washout |  | 0(0.00%) | 0(0.00%) | 1.000 |
| No washout |  | 31(100%) | 31(100%) |  |

NOTE: Data are represented in mean ± SD or frequency (%). And Data were evaluated by independent *t* test or Mann-Whitney *U* test for continuous variables and the Chi-square test or Fisher’s exact test for categorical variables; AP, [arterial](javascript:;) [phas](javascript:;)e; APHE, Peripheral arterial phase hyperenhancement

Figure E1 Changes in Treatment options After mpMR Imaging


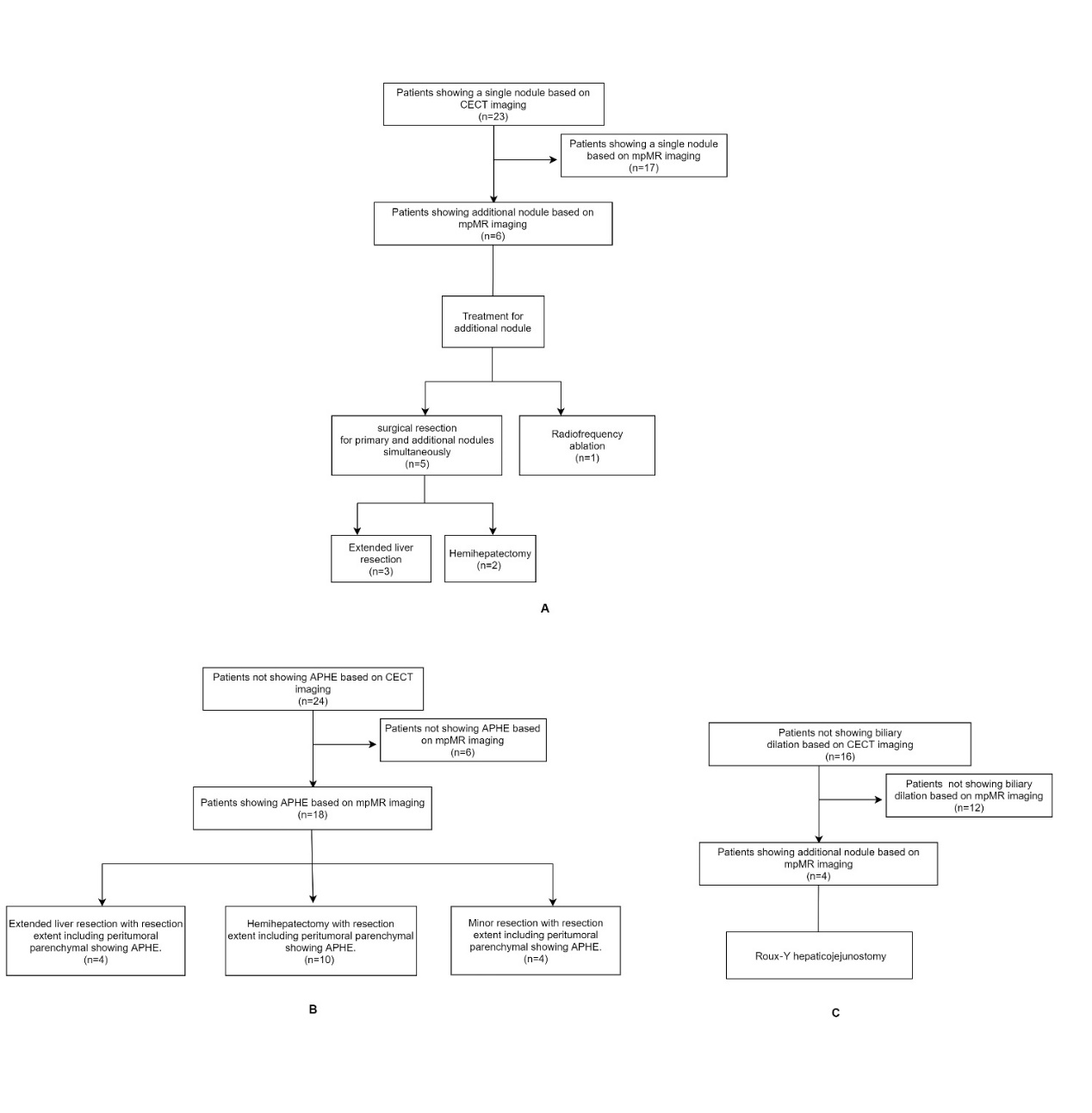


Figure E2 Scatter plot of (A) tumor size based on CT and
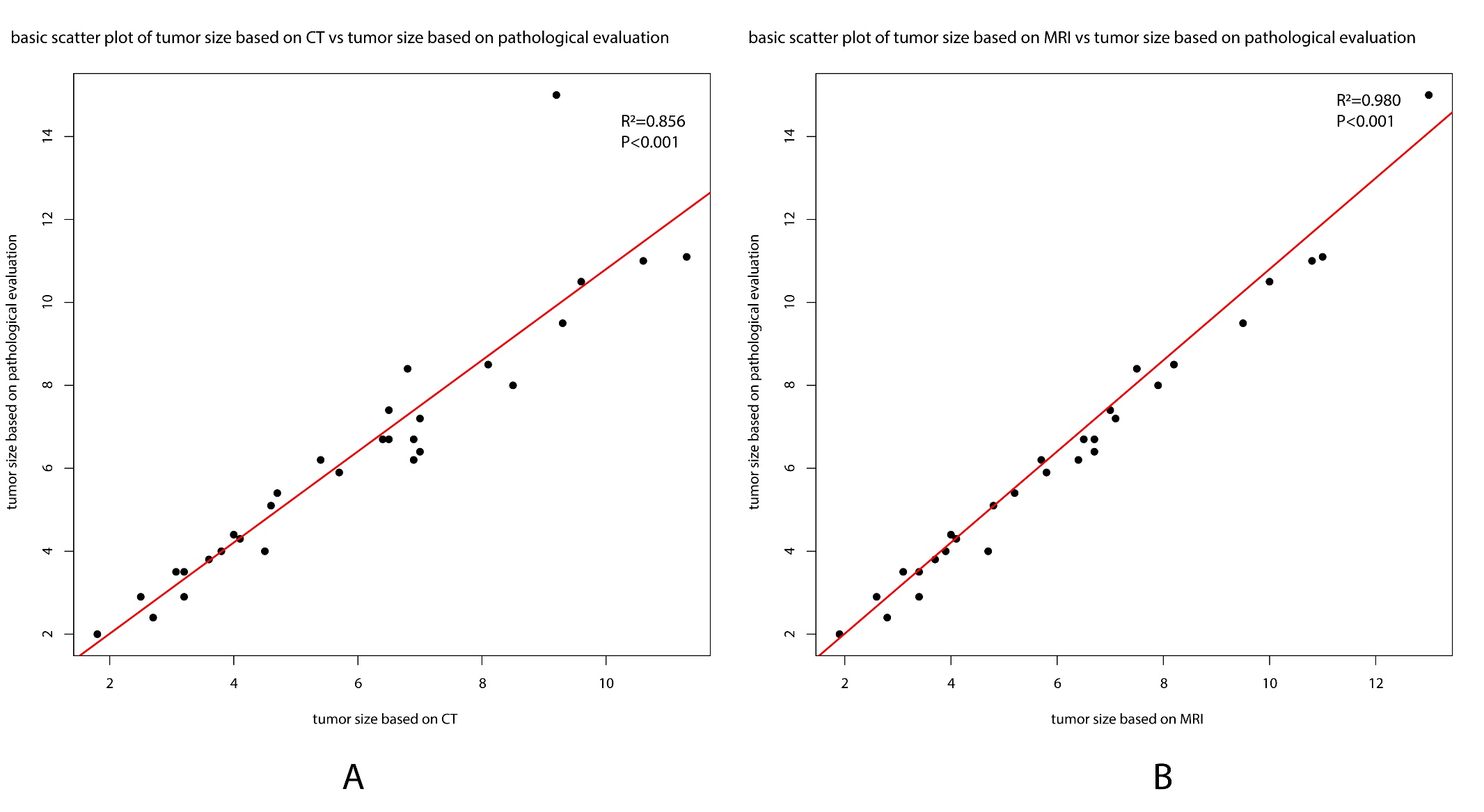
 tumor size based on MRI(B) vs pathological evaluation

Figure E3 Distribution of propensity scores before and after propensity score matching


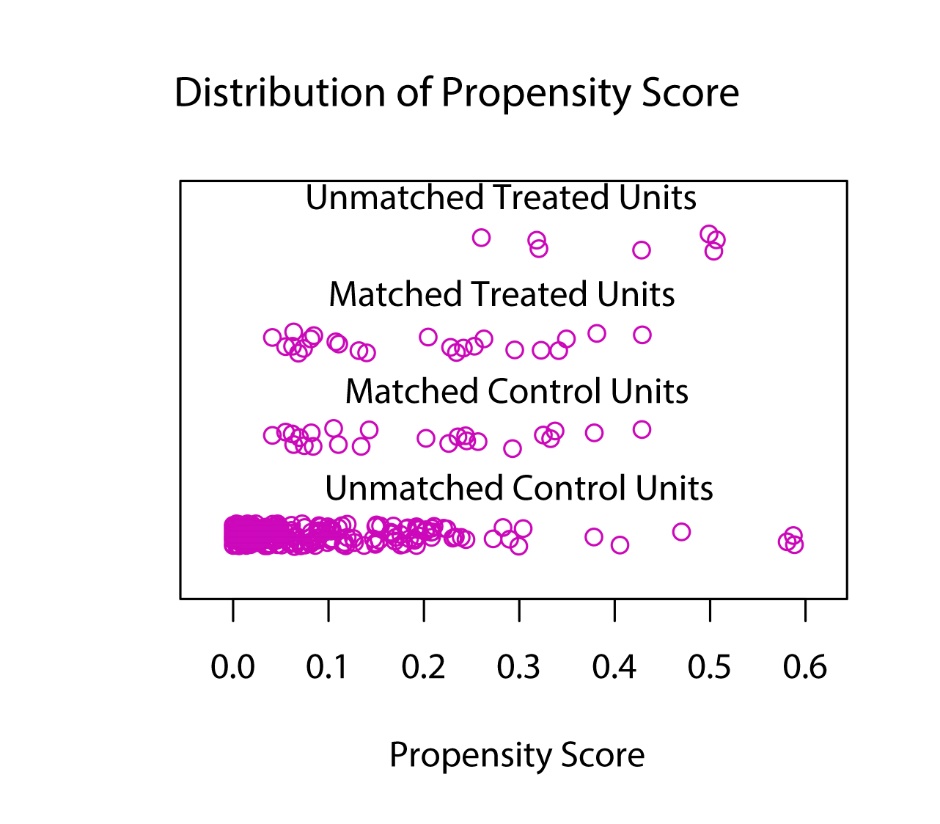


Figure E4 Kaplan-Meier curves of (A) overall survival and (B) recurrence-free rates of CT group and CT+MRI group after Propensity Score Matching


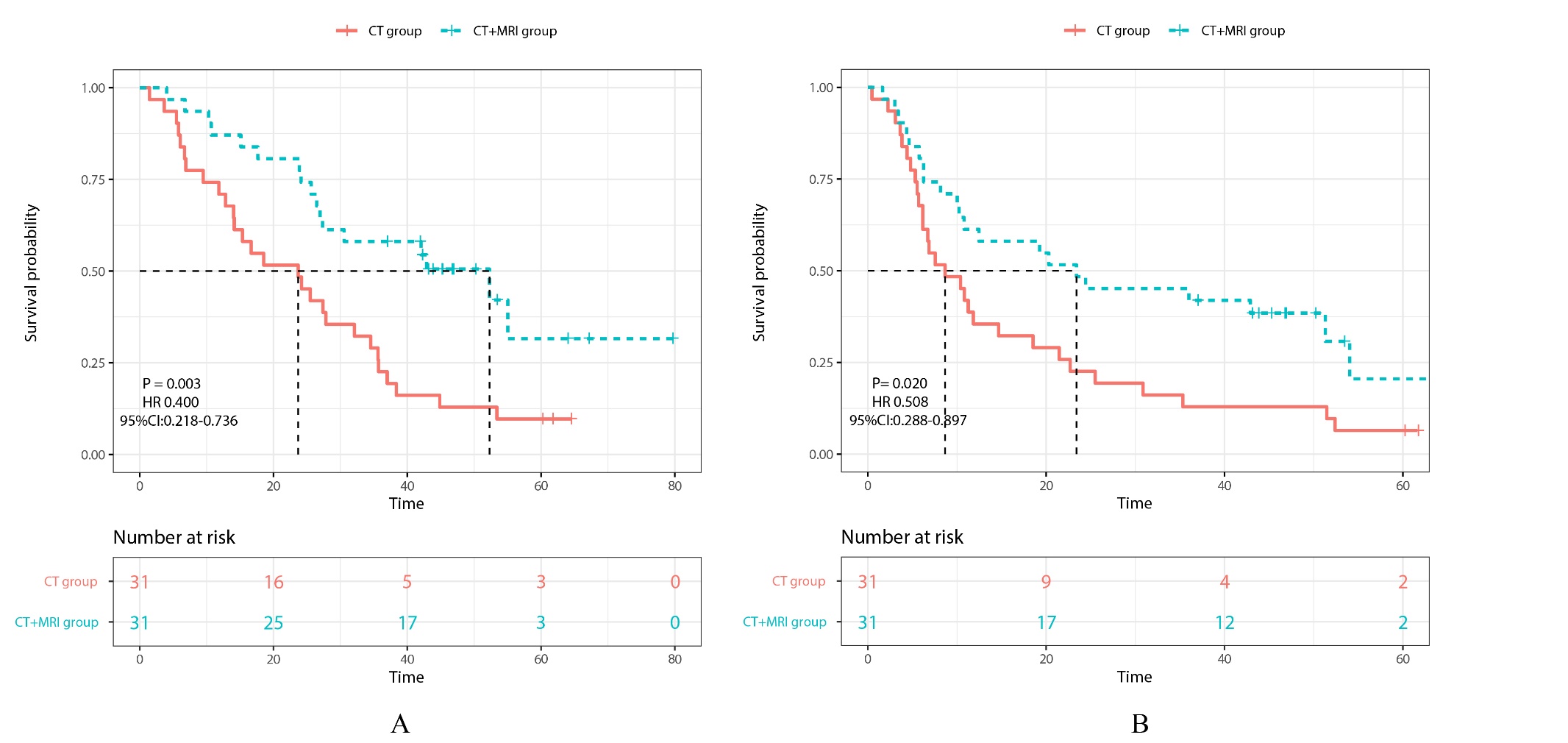

Supplement: Supplementary file 1 [file DataSheet_1.docx]
